# 5-Chloro-3-cyclo­pentyl­sulfonyl-2-methyl-1-benzofuran

**DOI:** 10.1107/S160053681103933X

**Published:** 2011-09-30

**Authors:** Pil Ja Seo, Hong Dae Choi, Byeng Wha Son, Uk Lee

**Affiliations:** a Department of Chemistry, Dongeui University, San 24 Kaya-dong Busanjin-gu, Busan 614-714, Republic of Korea; b Department of Chemistry, Pukyong National University, 599-1 Daeyeon 3-dong, Nam-gu, Busan 608-737, Republic of Korea

## Abstract

In the title compound, C_14_H_15_ClO_3_S, the cyclo­penyl ring adopts an envelope conformation. In the crystal, mol­ecules are linked by weak inter­molecular C—H⋯O hydrogen bonds into dual chains propagating in [100]. The dual chains arise from pairs of the same or different hydrogen bonds between adjacent molecules.

## Related literature

For the pharmacological activity of benzofuran compounds, see: Aslam *et al.* (2009 ▶); Galal *et al.* (2009 ▶); Khan *et al.* (2005 ▶). For natural products with benzofuran rings, see: Akgul & Anil (2003 ▶); Soekamto *et al.* (2003 ▶). For the crystal structures of related compounds, see: Seo *et al.* (2011*a* ▶,*b* ▶).
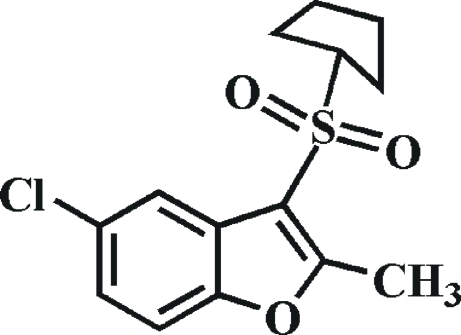


## Experimental

### 

#### Crystal data

C_14_H_15_ClO_3_S
*M* *_r_* = 298.77
Triclinic, 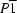

*a* = 7.4833 (8) Å
*b* = 8.7888 (9) Å
*c* = 10.9061 (10) Å
α = 66.919 (5)°
β = 82.848 (6)°
γ = 82.689 (6)°
*V* = 652.31 (11) Å^3^
*Z* = 2
Mo *K*α radiation
μ = 0.45 mm^−1^
*T* = 173 K
0.39 × 0.27 × 0.22 mm

#### Data collection

Bruker SMART APEXII CCD diffractometer
Absorption correction: multi-scan (*SADABS*; Bruker, 2009 ▶) *T*_min_ = 0.844, *T*_max_ = 0.905
11884 measured reflections
3252 independent reflections
2721 reflections with *I* > 2σ(*I*)
*R*_int_ = 0.046

#### Refinement

*R*[*F* ^2^ > 2σ(*F* ^2^)] = 0.044
*wR*(*F* ^2^) = 0.143
*S* = 1.05
3252 reflections
173 parameters
H-atom parameters constrained
Δρ_max_ = 0.44 e Å^−3^
Δρ_min_ = −0.73 e Å^−3^

### 

Data collection: *APEX2* (Bruker, 2009 ▶); cell refinement: *SAINT* (Bruker, 2009 ▶); data reduction: *SAINT*; program(s) used to solve structure: *SHELXS97* (Sheldrick, 2008 ▶); program(s) used to refine structure: *SHELXL97* (Sheldrick, 2008 ▶); molecular graphics: *ORTEP-3* (Farrugia, 1997 ▶) and *DIAMOND* (Brandenburg, 1998 ▶); software used to prepare material for publication: *SHELXL97*.

## Supplementary Material

Crystal structure: contains datablock(s) global, I. DOI: 10.1107/S160053681103933X/cv5153sup1.cif

Structure factors: contains datablock(s) I. DOI: 10.1107/S160053681103933X/cv5153Isup2.hkl

Supplementary material file. DOI: 10.1107/S160053681103933X/cv5153Isup3.cml

Additional supplementary materials:  crystallographic information; 3D view; checkCIF report

## Figures and Tables

**Table 1:** Hydrogen-bond geometry (Å, °)

*D*—H⋯*A*	*D*—H	H⋯*A*	*D*⋯*A*	*D*—H⋯*A*
C3—H3⋯O3^i^	0.95	2.51	3.420 (2)	160
C12—H12*A*⋯O2^ii^	0.99	2.59	3.557 (2)	167
C13—H13*B*⋯O3^ii^	0.99	2.61	3.516 (3)	153

Symmetry codes: (i) 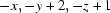; (ii) 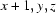.

**Fig. 1.**
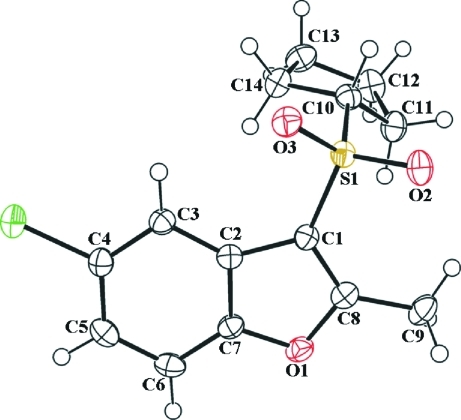
The molecular structure of the title compound with the atom numbering scheme. Displacement ellipsoids are drawn at the 50% probability level. H atoms are presented as small spheres of arbitrary radius.

**Fig. 2.**
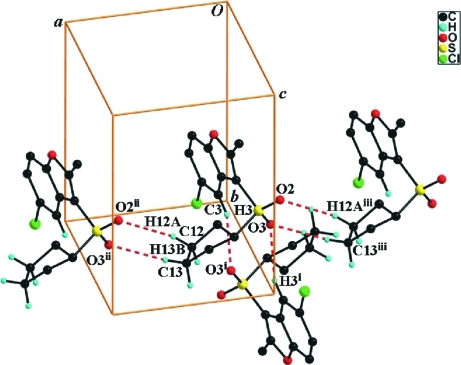
A view of the C—H···O hydrogen bonds (dotted lines) in the crystal structure of the title compound [symmetry codes: (i) - x, - y + 2, - z + 1; (ii) x + 1, y, z; (iii) x - 1, y, z.] H atoms non-participating in hydrogen-bonding were omitted for clarity.

## supplementary crystallographic information

### Comment

Recently, many compounds containing a benzofuran moiety have received much attention because of their valuable pharmacological properties such as antibacterial and antifungal, antitumor and antiviral, and antimicrobial activities (Aslam *et al.*, 2009, Galal *et al.*, 2009, Khan *et al.*, 2005). These benzofuran derivatives occur in a wide range of natural products (Akgul & Anil, 2003; Soekamto *et al.*, 2003). As a part of our ongoing study of benzofuran derivatives containing either 3-cyclopentylsulfinyl (Seo *et al.*, 2011*a*) or 3-cyclopentylsulfonyl (Seo *et al.*, 2011*b*) substituents, we report herein the crystal structure of the title compound (I).

In (I) (Fig. 1), the benzofuran unit is essentially planar, with a mean deviation of 0.005 (2) Å from the least-squares plane defined by the nine constituent atoms. The cyclopentyl ring is in the envelope form. In the crystal structure (Fig. 2), weak intermolecular C—H···O hydrogen bonds (Table 1) link molecules into dual chains propagated in [100] (Fig. 2).

### Experimental

77% 3-Chloroperoxybenzoic acid (560 mg, 2.5 mmol) was added in small portions to a stirred solution of 5-chloro-3-cyclopentylsulfanyl-2-methyl-1-benzofuran (320 mg, 1.2 mmol) in dichloromethane (40 mL) at 273 K. After being stirred at room temperature for 8h, the mixture was washed with saturated sodium bicarbonate solution and the organic layer was separated, dried over magnesium sulfate, filtered and concentrated at reduced pressure. The residue was purified by column chromatography (hexane–ethyl acetate, 4:1 v/v) to afford the title compound as a colorless solid [yield 70%, m.p. 412–413 K; *R*_f_ = 0.46 (hexane–ethyl acetate, 4:1 v/v)]. Single crystals suitable for X-ray diffraction were prepared by slow evaporation of a solution of the title compound in acetone at room temperature.

### Refinement

All H atoms were positioned geometrically and refined using a riding model, with C—H = 0.95 Å for aryl, 1.00 Å for methine, 0.99 Å for methylene and 0.98 Å for methyl H atoms, respectively. *U*_iso_(H) =1.2*U*_eq_(C) for aryl,  methine and methylene, and 1.5*U*_eq_(C)  for methyl H atoms.

### Crystal data

**Table:**

C_14_H_15_ClO_3_S	*Z* = 2
*M**_r_* = 298.77	*F*(000) = 312
Triclinic, *P*1	*D*_x_ = 1.521 Mg m^−^^3^
Hall symbol: -P 1	Mo *K*α radiation, λ = 0.71073 Å
*a* = 7.4833 (8) Å	Cell parameters from 5081 reflections
*b* = 8.7888 (9) Å	θ = 2.5–28.2°
*c* = 10.9061 (10) Å	µ = 0.45 mm^−^^1^
α = 66.919 (5)°	*T* = 173 K
β = 82.848 (6)°	Block, colourless
γ = 82.689 (6)°	0.39 × 0.27 × 0.22 mm
*V* = 652.31 (11) Å^3^	

### Data collection

**Table:**

Bruker SMART APEXII CCD diffractometer	3252 independent reflections
Radiation source: rotating anode	2721 reflections with *I* > 2σ(*I*)
graphite multilayer	*R*_int_ = 0.046
Detector resolution: 10.0 pixels mm^-1^	θ_max_ = 28.4°, θ_min_ = 2.0°
φ and ω scans	*h* = −9→9
Absorption correction: multi-scan (*SADABS*; Bruker, 2009)	*k* = −11→11
*T*_min_ = 0.844, *T*_max_ = 0.905	*l* = −14→14
11884 measured reflections	

### Refinement

**Table:**

Refinement on *F*^2^	Primary atom site location: structure-invariant direct methods
Least-squares matrix: full	Secondary atom site location: difference Fourier map
*R*[*F*^2^ > 2σ(*F*^2^)] = 0.044	Hydrogen site location: difference Fourier map
*wR*(*F*^2^) = 0.143	H-atom parameters constrained
*S* = 1.05	*w* = 1/[σ^2^(*F*_o_^2^) + (0.1*P*)^2^] where *P* = (*F*_o_^2^ + 2*F*_c_^2^)/3
3252 reflections	(Δ/σ)_max_ = 0.001
173 parameters	Δρ_max_ = 0.44 e Å^−^^3^
0 restraints	Δρ_min_ = −0.73 e Å^−^^3^

### Special details

**Table:**

Geometry. All esds (except the esd in the dihedral angle between two l.s. planes) are estimated using the full covariance matrix. The cell esds are taken into account individually in the estimation of esds in distances, angles and torsion angles; correlations between esds in cell parameters are only used when they are defined by crystal symmetry. An approximate (isotropic) treatment of cell esds is used for estimating esds involving l.s. planes.
Refinement. Refinement of F^2^ against ALL reflections. The weighted R-factor wR and goodness of fit S are based on F^2^, conventional R-factors R are based on F, with F set to zero for negative F^2^. The threshold expression of F^2^ > 2sigma(F^2^) is used only for calculating R-factors(gt) etc. and is not relevant to the choice of reflections for refinement. R-factors based on F^2^ are statistically about twice as large as those based on F, and R- factors based on ALL data will be even larger.

### Fractional atomic coordinates and isotropic or equivalent isotropic displacement parameters (Å^2^)

**Table:**

	*x*	*y*	*z*	*U*_iso_*/*U*_eq_	
S1	0.01806 (6)	0.70534 (5)	0.73025 (4)	0.02070 (15)	
Cl1	0.22626 (7)	0.92775 (6)	0.13290 (4)	0.03364 (17)	
O1	0.26706 (18)	0.34648 (14)	0.62681 (11)	0.0261 (3)	
O2	−0.07756 (18)	0.60437 (16)	0.85224 (11)	0.0291 (3)	
O3	−0.08387 (17)	0.83205 (15)	0.62857 (11)	0.0257 (3)	
C1	0.1369 (2)	0.5756 (2)	0.65566 (15)	0.0210 (3)	
C2	0.1864 (2)	0.6199 (2)	0.51383 (15)	0.0209 (3)	
C3	0.1721 (2)	0.7648 (2)	0.39920 (15)	0.0225 (4)	
H3	0.1194	0.8673	0.4030	0.027*	
C4	0.2388 (3)	0.7506 (2)	0.28003 (16)	0.0243 (4)	
C5	0.3159 (3)	0.6020 (2)	0.27022 (17)	0.0276 (4)	
H5	0.3586	0.5994	0.1853	0.033*	
C6	0.3301 (3)	0.4594 (2)	0.38371 (17)	0.0274 (4)	
H6	0.3818	0.3568	0.3797	0.033*	
C7	0.2654 (2)	0.4727 (2)	0.50369 (15)	0.0221 (4)	
C8	0.1883 (2)	0.4117 (2)	0.71738 (16)	0.0245 (4)	
C9	0.1791 (3)	0.2926 (2)	0.85921 (17)	0.0321 (4)	
H9A	0.1229	0.3503	0.9169	0.048*	
H9B	0.3015	0.2468	0.8848	0.048*	
H9C	0.1068	0.2023	0.8693	0.048*	
C10	0.1818 (2)	0.8010 (2)	0.77215 (15)	0.0232 (4)	
H10	0.1163	0.8755	0.8158	0.028*	
C11	0.3143 (3)	0.6800 (2)	0.86896 (17)	0.0295 (4)	
H11A	0.2577	0.6388	0.9618	0.035*	
H11B	0.3568	0.5842	0.8436	0.035*	
C12	0.4688 (3)	0.7835 (3)	0.85509 (18)	0.0328 (4)	
H12A	0.5859	0.7157	0.8595	0.039*	
H12B	0.4556	0.8253	0.9281	0.039*	
C13	0.4615 (3)	0.9290 (2)	0.71898 (19)	0.0327 (4)	
H13A	0.4423	1.0361	0.7310	0.039*	
H13B	0.5758	0.9276	0.6629	0.039*	
C14	0.3028 (3)	0.9064 (2)	0.65330 (16)	0.0278 (4)	
H14A	0.2385	1.0147	0.6028	0.033*	
H14B	0.3446	0.8483	0.5920	0.033*	

### Atomic displacement parameters (Å^2^)

**Table:**

	*U*^11^	*U*^22^	*U*^33^	*U*^12^	*U*^13^	*U*^23^
S1	0.0169 (3)	0.0228 (2)	0.0204 (2)	0.00049 (18)	−0.00042 (16)	−0.00706 (17)
Cl1	0.0423 (3)	0.0320 (3)	0.0211 (2)	−0.0046 (2)	−0.00045 (19)	−0.00445 (19)
O1	0.0293 (8)	0.0185 (6)	0.0285 (6)	0.0010 (5)	−0.0056 (5)	−0.0068 (5)
O2	0.0240 (7)	0.0365 (7)	0.0233 (6)	−0.0060 (6)	0.0040 (5)	−0.0084 (5)
O3	0.0214 (7)	0.0259 (6)	0.0272 (6)	0.0044 (5)	−0.0051 (5)	−0.0082 (5)
C1	0.0198 (9)	0.0212 (8)	0.0210 (7)	−0.0016 (7)	−0.0013 (6)	−0.0070 (6)
C2	0.0180 (9)	0.0215 (8)	0.0235 (7)	−0.0010 (7)	−0.0017 (6)	−0.0092 (6)
C3	0.0212 (9)	0.0217 (8)	0.0238 (7)	−0.0002 (7)	−0.0019 (6)	−0.0085 (6)
C4	0.0233 (10)	0.0263 (8)	0.0218 (7)	−0.0041 (7)	−0.0018 (6)	−0.0071 (6)
C5	0.0243 (10)	0.0352 (10)	0.0276 (8)	−0.0022 (8)	−0.0002 (7)	−0.0175 (8)
C6	0.0263 (10)	0.0258 (8)	0.0350 (9)	0.0027 (8)	−0.0045 (7)	−0.0176 (7)
C7	0.0209 (9)	0.0193 (7)	0.0250 (7)	−0.0007 (7)	−0.0041 (6)	−0.0070 (6)
C8	0.0221 (10)	0.0233 (8)	0.0272 (8)	−0.0024 (7)	−0.0039 (7)	−0.0079 (7)
C9	0.0375 (12)	0.0222 (8)	0.0295 (8)	−0.0025 (8)	−0.0084 (8)	−0.0007 (7)
C10	0.0202 (9)	0.0247 (8)	0.0245 (7)	0.0015 (7)	−0.0009 (6)	−0.0105 (7)
C11	0.0276 (11)	0.0326 (9)	0.0252 (8)	−0.0018 (8)	−0.0059 (7)	−0.0068 (7)
C12	0.0241 (11)	0.0447 (11)	0.0293 (9)	−0.0041 (9)	−0.0041 (7)	−0.0129 (8)
C13	0.0289 (12)	0.0274 (9)	0.0411 (10)	−0.0038 (8)	−0.0058 (8)	−0.0109 (8)
C14	0.0267 (11)	0.0265 (8)	0.0266 (8)	−0.0038 (8)	−0.0033 (7)	−0.0054 (7)

### Geometric parameters (Å, °)

**Table:**

S1—O2	1.4382 (12)	C8—C9	1.488 (2)
S1—O3	1.4415 (11)	C9—H9A	0.9800
S1—C1	1.7441 (16)	C9—H9B	0.9800
S1—C10	1.7664 (18)	C9—H9C	0.9800
Cl1—C4	1.7463 (17)	C10—C14	1.529 (2)
O1—C8	1.365 (2)	C10—C11	1.530 (2)
O1—C7	1.3662 (18)	C10—H10	1.0000
C1—C8	1.356 (2)	C11—C12	1.517 (3)
C1—C2	1.450 (2)	C11—H11A	0.9900
C2—C7	1.392 (2)	C11—H11B	0.9900
C2—C3	1.395 (2)	C12—C13	1.535 (3)
C3—C4	1.380 (2)	C12—H12A	0.9900
C3—H3	0.9500	C12—H12B	0.9900
C4—C5	1.397 (3)	C13—C14	1.531 (3)
C5—C6	1.378 (2)	C13—H13A	0.9900
C5—H5	0.9500	C13—H13B	0.9900
C6—C7	1.381 (2)	C14—H14A	0.9900
C6—H6	0.9500	C14—H14B	0.9900
			
O2—S1—O3	118.65 (8)	C8—C9—H9C	109.5
O2—S1—C1	108.48 (8)	H9A—C9—H9C	109.5
O3—S1—C1	106.51 (7)	H9B—C9—H9C	109.5
O2—S1—C10	108.07 (8)	C14—C10—C11	104.30 (15)
O3—S1—C10	108.11 (8)	C14—C10—S1	114.73 (12)
C1—S1—C10	106.39 (8)	C11—C10—S1	114.46 (12)
C8—O1—C7	106.90 (12)	C14—C10—H10	107.7
C8—C1—C2	107.01 (14)	C11—C10—H10	107.7
C8—C1—S1	126.67 (12)	S1—C10—H10	107.7
C2—C1—S1	126.15 (12)	C12—C11—C10	103.74 (15)
C7—C2—C3	120.01 (14)	C12—C11—H11A	111.0
C7—C2—C1	104.43 (14)	C10—C11—H11A	111.0
C3—C2—C1	135.56 (15)	C12—C11—H11B	111.0
C4—C3—C2	116.08 (15)	C10—C11—H11B	111.0
C4—C3—H3	122.0	H11A—C11—H11B	109.0
C2—C3—H3	122.0	C11—C12—C13	107.18 (15)
C3—C4—C5	123.68 (16)	C11—C12—H12A	110.3
C3—C4—Cl1	118.36 (13)	C13—C12—H12A	110.3
C5—C4—Cl1	117.96 (13)	C11—C12—H12B	110.3
C6—C5—C4	120.02 (15)	C13—C12—H12B	110.3
C6—C5—H5	120.0	H12A—C12—H12B	108.5
C4—C5—H5	120.0	C14—C13—C12	106.39 (17)
C5—C6—C7	116.75 (15)	C14—C13—H13A	110.5
C5—C6—H6	121.6	C12—C13—H13A	110.5
C7—C6—H6	121.6	C14—C13—H13B	110.5
O1—C7—C6	125.78 (14)	C12—C13—H13B	110.5
O1—C7—C2	110.78 (13)	H13A—C13—H13B	108.6
C6—C7—C2	123.44 (15)	C10—C14—C13	103.38 (14)
C1—C8—O1	110.88 (14)	C10—C14—H14A	111.1
C1—C8—C9	134.00 (15)	C13—C14—H14A	111.1
O1—C8—C9	115.11 (14)	C10—C14—H14B	111.1
C8—C9—H9A	109.5	C13—C14—H14B	111.1
C8—C9—H9B	109.5	H14A—C14—H14B	109.1
H9A—C9—H9B	109.5		
			
O2—S1—C1—C8	−22.22 (19)	C1—C2—C7—O1	−0.2 (2)
O3—S1—C1—C8	−151.00 (17)	C3—C2—C7—C6	−1.1 (3)
C10—S1—C1—C8	93.83 (18)	C1—C2—C7—C6	179.06 (17)
O2—S1—C1—C2	152.39 (16)	C2—C1—C8—O1	0.0 (2)
O3—S1—C1—C2	23.61 (18)	S1—C1—C8—O1	175.43 (13)
C10—S1—C1—C2	−91.55 (17)	C2—C1—C8—C9	179.0 (2)
C8—C1—C2—C7	0.1 (2)	S1—C1—C8—C9	−5.5 (3)
S1—C1—C2—C7	−175.35 (14)	C7—O1—C8—C1	−0.1 (2)
C8—C1—C2—C3	−179.7 (2)	C7—O1—C8—C9	−179.37 (15)
S1—C1—C2—C3	4.8 (3)	O2—S1—C10—C14	176.93 (11)
C7—C2—C3—C4	0.3 (3)	O3—S1—C10—C14	−53.47 (13)
C1—C2—C3—C4	−179.85 (19)	C1—S1—C10—C14	60.60 (13)
C2—C3—C4—C5	0.5 (3)	O2—S1—C10—C11	56.38 (14)
C2—C3—C4—Cl1	−179.62 (13)	O3—S1—C10—C11	−174.02 (12)
C3—C4—C5—C6	−0.6 (3)	C1—S1—C10—C11	−59.95 (13)
Cl1—C4—C5—C6	179.48 (15)	C14—C10—C11—C12	37.10 (17)
C4—C5—C6—C7	−0.1 (3)	S1—C10—C11—C12	163.27 (12)
C8—O1—C7—C6	−179.05 (19)	C10—C11—C12—C13	−22.06 (19)
C8—O1—C7—C2	0.2 (2)	C11—C12—C13—C14	−1.1 (2)
C5—C6—C7—O1	−179.91 (17)	C11—C10—C14—C13	−37.65 (17)
C5—C6—C7—C2	0.9 (3)	S1—C10—C14—C13	−163.66 (12)
C3—C2—C7—O1	179.66 (15)	C12—C13—C14—C10	23.73 (18)

### Hydrogen-bond geometry (Å, °)

**Table:**

*D*—H···*A*	*D*—H	H···*A*	*D*···*A*	*D*—H···*A*
C3—H3···O3^i^	0.95	2.51	3.420 (2)	160.
C12—H12A···O2^ii^	0.99	2.59	3.557 (2)	167.
C13—H13B···O3^ii^	0.99	2.61	3.516 (3)	153.

Symmetry codes: (i) −*x*, −*y*+2, −*z*+1; (ii) *x*+1, *y*, *z*.
